# Ecophysiological consequences of alcoholism on human gut microbiota: implications for ethanol-related pathogenesis of colon cancer

**DOI:** 10.1038/srep27923

**Published:** 2016-06-13

**Authors:** Atsuki Tsuruya, Akika Kuwahara, Yuta Saito, Haruhiko Yamaguchi, Takahisa Tsubo, Shogo Suga, Makoto Inai, Yuichi Aoki, Seiji Takahashi, Eri Tsutsumi, Yoshihide Suwa, Hidetoshi Morita, Kenji Kinoshita, Yukari Totsuka, Wataru Suda, Kenshiro Oshima, Masahira Hattori, Takeshi Mizukami, Akira Yokoyama, Takefumi Shimoyama, Toru Nakayama

**Affiliations:** 1 Department of Biomolecular Engineering, Graduate School of Engineering, Tohoku University, Sendai, Miyagi 980-8579 Japan; 2 Department of Applied Information Sciences, Graduate School of Information Sciences, Tohoku University, Sendai, Miyagi 980-8579 Japan; 3 Suntory World Research Center, Suntory Holdings Ltd., Soraku-gun, Kyoto 619-0284, Japan; 4 Graduate School of Environmental and Life Science, Okayama University, 1-1-1 Tsushima-naka, Kita-ku, Okayama 700-8530, Japan; 5 School of Pharmaceutical Sciences, Mukogawa Women’s University, Nishinomiya, Hyogo 663-8179, Japan; 6 Division of Cancer Development System, National Cancer Center Research Institute, Chuo-ku, Tokyo 104-0045, Japan; 7 Center for Omics and Bioinformatics, Graduate School of Frontier Sciences, The University of Tokyo, Kashiwa, Chiba 277-8561, Japan; 8 National Hospital Organization Kurihama Medical and Addiction Center, Yokosuka, Kanagawa 239-0841, Japan

## Abstract

Chronic consumption of excess ethanol increases the risk of colorectal cancer. The pathogenesis of ethanol-related colorectal cancer (ER-CRC) is thought to be partly mediated by gut microbes. Specifically, bacteria in the colon and rectum convert ethanol to acetaldehyde (AcH), which is carcinogenic. However, the effects of chronic ethanol consumption on the human gut microbiome are poorly understood, and the role of gut microbes in the proposed AcH-mediated pathogenesis of ER-CRC remains to be elaborated. Here we analyse and compare the gut microbiota structures of non-alcoholics and alcoholics. The gut microbiotas of alcoholics were diminished in dominant obligate anaerobes (*e.g*., *Bacteroides* and *Ruminococcus*) and enriched in *Streptococcus* and other minor species. This alteration might be exacerbated by habitual smoking. These observations could at least partly be explained by the susceptibility of obligate anaerobes to reactive oxygen species, which are increased by chronic exposure of the gut mucosa to ethanol. The AcH productivity from ethanol was much lower in the faeces of alcoholic patients than in faeces of non-alcoholic subjects. The faecal phenotype of the alcoholics could be rationalised based on their gut microbiota structures and the ability of gut bacteria to accumulate AcH from ethanol.

Chronic consumption of excess ethanol through habitual heavy drinking is a risk factor for several cancers, including colorectal cancer (CRC)^1^,^2^. Indeed, the risk of ethanol-related CRC (ER-CRC) might be extremely high in alcoholics. For example, recent colonoscopic screening results of Japanese alcoholic men revealed colorectal adenoma in 54.5% of these subjects, and intramucosal and invasive CRCs in 5.9%^3^. The mechanism by which chronic ethanol consumption increases the CRC risk has not been established^2^, although commensal gut bacteria are proposed to be partly involved in ER-CRC pathogenesis^2^,^4^.

In living systems, ethanol induces the formation of reactive oxygen species (ROS), which promote oxidative stress via a variety of cellular processes^5^,^6^. A role for oxidative stress in the pathogenesis of human carcinogenesis (including CRC) has been proposed^7^. The mucosal and bacterial pathway of ethanol oxidation under the aerobic conditions of the colon and rectum has also been implicated in ER-CRC pathogenesis^4^,^8^,^9^,^10^. The first metabolite of ethanol oxidation in these pathways is acetaldehyde (AcH), a mutagen with an estimated minimum mutagenic concentration (MMC) of 50–150 μM^11^,^12^. Recently, AcH associated with alcohol consumption has been revealed to be carcinogenic to humans^13^. Thus, the oxidation of ethanol in the colon and rectum might increase the ER-CRC risk by enhancing the intracolorectal levels of AcH above the MMC^2^,^4^. Previous studies showed that, in addition to colorectal mucosal cells, intestinal aerobes and facultative anaerobes play important roles in the production of AcH from ethanol under aerobic conditions in the colon and rectum (namely, the bacteriocolonic pathway of ethanol oxidation)^14^,^15^,^16^. Moreover, we recently identified *Ruminococcus* and several obligate anaerobes (*Collinsella*, *Prevotella*, *Coriobacterium*, and *Bifidobacterium*), which are major forms of faecal bacteria, as important potential AcH accumulators in the colon and rectum^17^.

The structure of human gut microbiota (GM; *i.e*., the commensal microbial communities) is altered by diet and various disease states^18^,^19^,^20^. By elucidating how these factors influence the structural characteristics of the GM, we may acquire strategic hints for maintaining human health and for diagnosing, treating, and even preventing diseases^20^. Thus far, how the chronic consumption of large amounts of ethanol affects human GM structures and the bacteriocolonic pathway of ethanol oxidation (see above) is not known, although the GM structures and ethanol metabolism of intestinal bacteria might be related to ER-CRC pathogenesis. To better understand these issues, a basic investigation using bacterial ecology and physiology approaches is needed. Because alcoholics have been habitual heavy drinkers for many years, and ethanol constitutes a main component of their diets, the faecal samples of alcoholics should reveal the effects of chronic ethanol consumption on the human GM structure. Mutlu *et al.*^21^ analysed the structures of the colonic mucosa-associated microbiota of alcoholics, and reported differences from those of healthy subjects at the phylum level. The following year, Bull-Otterson *et al.*^22^ reported the temporal effects of chronic ethanol consumption on the GM structure in a mouse model. However, to properly understand the effects of chronic ethanol consumption on the human GM structure in conjunction with the pathogenesis of ER-CRC, we require the genus-level details of human GM structures.

In this study, to clarify the ecophysiological consequences of chronic ethanol consumption (alcoholism) on human GM structures, we compare the GM structures of Japanese alcoholic patients and non-alcoholic volunteers. The GM of alcoholics showed more phylogenetic diversity (β-diversity) than those of non-alcoholics, with a diminution of dominant obligate anaerobes such as *Bacteroides*, *Bifidobacterium*, and *Ruminococcus* and an enrichment of *Streptococcus* and many other minor bacterial species. This finding might be at least partially explained by the intestinal oxidative stress induced by chronic ethanol exposure, to which obligate anaerobes are susceptible. The faeces of the alcoholic patients produced much less AcH from ethanol than the faeces of non-alcoholics, and the faecal phenotypes of these patients could be consistently rationalised by their altered GM structures. These observations provide important information for understanding the mechanisms of ER-CRC pathogenesis.

## Results

### GM analysis

The microbiota of the faeces obtained from 16 alcoholic patients were characterised by 454 barcoded pyrosequencing (AL02–AL18; Supplementary Table S1). The results were compared with those of 48 healthy subjects (non-alcoholics, NA01–NA48; Supplementary Table S1).

**Figure 1:**
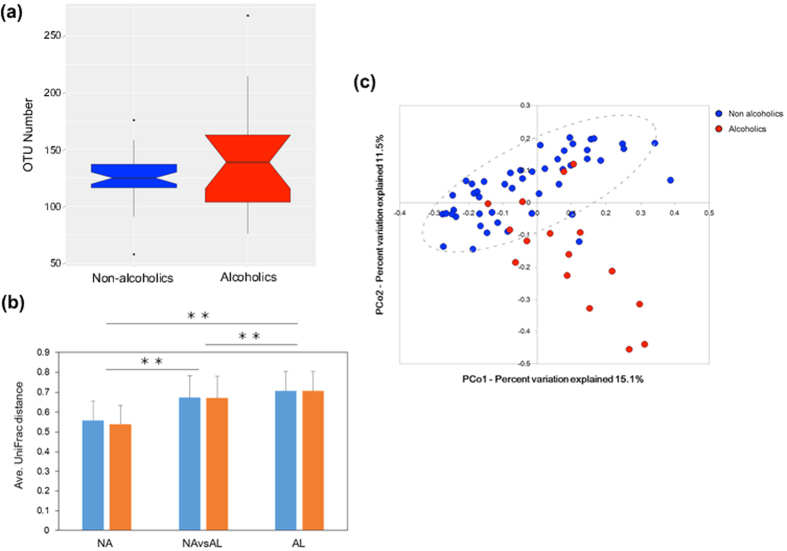
OTU and UniFrac principal coordinate analyses of faecal bacterial communities of alcoholic patients (*n* = 16) and non-alcoholic volunteers (*n* = 48). (**a**) Notched box plots of faecal OTU numbers (α-diversity). (**b**) Weighted average UniFrac distances within the non-alcoholic group (NA, *blue bars*), within the alcoholic group (AL, *blue bars*), and between these two groups (NA vs. AL, *blue bar*). Weighted average UniFrac distances were also determined within the non-alcoholic men (i.e., excluding women; NA, *orange bars*; *n* = 28), within the alcoholic men (AL, *orange bars*; *n* = 16), and between these two groups (NA vs. AL, *orange bars*). **P < 0.05 (Welch’s *t-*test with 10,000 Monte Carlo simulations, adjusted based on the Bonferroni procedure). (**c**) Faecal bacterial communities in the alcoholic patients (*red symbols*) and non-alcoholic volunteers (*blue symbols)* were clustered by PCoA of the weighted UniFrac distance matrix. In the PCoA, PCo1 and PCo2 explained 15.1% and 11.5% of the variation, respectively. *Grey oval* (*dashed line*) delineates a possible cluster of the non-alcoholic GM structures.

We analysed the species richness (α-diversity) in the faecal microbial communities of both groups by comparing the number of species-level operational taxonomic units (OTUs) among the individuals, defined as the number of clusters sharing ≥96% sequence identity. The alcoholic group showed greater inter-member variability in its OTU numbers than the non-alcoholic group (Fig. 1(a)). We then analysed the phylogenetic diversity among the GM structures (i.e., the β-diversity) using multivariate methods (namely, principal coordinate analysis; PCoA)^23^. The PCoA matrices contained the phylogenetically weighted distances (the unique fraction metric or UniFrac^23^) between all combinations of subjects. The average weighted UniFrac distances quantify the phylogenetic β-diversity between groups; a group with a smaller UniFrac distance has a lower phylogenetic variation (i.e., smaller β-diversity) in its GM structure. The average UniFrac distance was larger among the alcoholic samples than among the non-alcoholic samples and between the alcoholic and non-alcoholic samples (Fig. 1(b), *blue bars*). Moreover, the difference between any pair of these three distances was statistically significant. These results indicate that the GM structures were significantly more diverse in the alcoholics than in the non-alcoholics. The UniFrac-PCoA analyses revealed different clusterings of the GM structures in the alcoholic patients and non-alcoholic participants (Fig. 1(c)), suggesting that alcoholism alters the GM structures from those generally observed in non-alcoholics. Note that the 48 non-alcoholic subjects included both men and women, and covered a wide generational range [young (15–30 years old), mature (31–44 years old), and middle-aged (45–65 years old)]. The UniFrac-PCoA plots of this group appeared to form a cluster (enclosed by the *dashed oval* in Fig. 1(c)), which likely represents the range of possible variation in their GM structures (with two exceptions), irrespective of sex or generation. By contrast, the recruited alcoholic patients were all male, and most of them were mature or middle-aged. Despite the single sex and limited generational variation of the alcoholic subjects, the 16 alcoholic plots were scattered across the UniFrac-PCoA diagram, and 11 of them were outside the non-alcoholic cluster. Such diversity suggests disordered patterns in the GM (i.e., dysbiosis) of the alcoholic patients. The segregation of the GM structures in alcoholics and non-alcoholics remained when women and youth were excluded from the comparisons (see also Fig. 1(b), *orange bars*).

To characterise the dysbiotic nature of the GM in the alcoholic patients, we analysed the GM phylogenies of both alcoholic and non-alcoholic groups at the phylum and genus levels. In order of decreasing abundance, the common phyla in the GM of non-alcoholics were *Firmicutes*, *Bacteroidetes*, *Actinobacteria*, *Proteobacteria*, and *Fusobacteria* (Supplementary Fig. S1), consistent with the previously proposed general consensus of phylum-level human gut flora^20^,^24^. Although the same phyla were found in the guts of alcoholic patients, *Bacteroidetes* formed a significantly smaller proportion of the GM than in non-alcoholics (*P*-value < 0.01) (Supplementary Fig. S2). The relative abundance of *Proteobacteria* appeared to be increased in alcoholics, but the difference was not statistically significant (*P*-value > 0.05).

**Figure 2:**
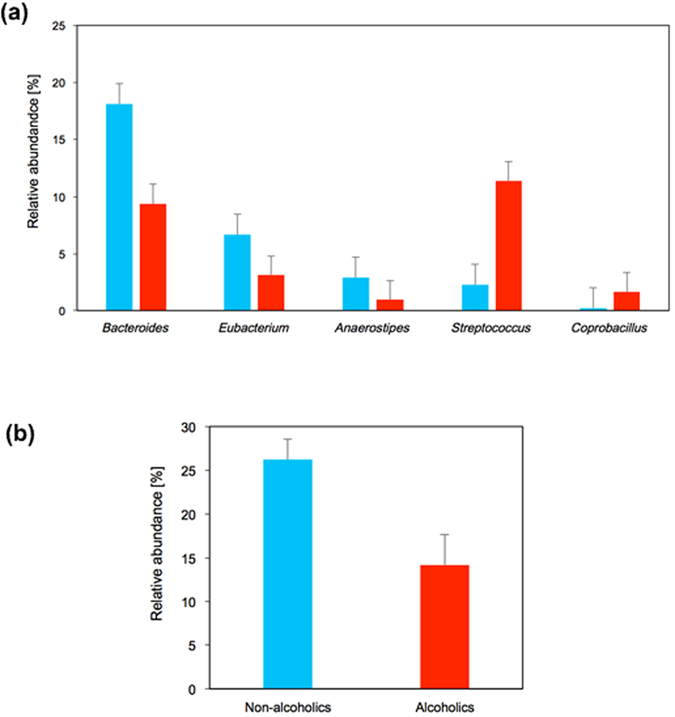
Comparison of relative abundances of bacteria between the faecal bacterial communities of the alcoholic and non-alcoholic groups. (**a**) Relative abundances of bacterial genera showing significant differences (*P* < 0.05; Welch test). (**b**) Relative abundances of obligate anaerobes that potentially accumulate AcH to high levels (*i.e.*, *Ruminococcus*, *Bifidobacterium*, *Collinsella*, and *Prevotella*)^17^. *Blue bars*, non-alcoholic group; *Red bars*, alcoholic group. *P* < 0.01.

Figure 2(a) shows the relative abundance of bacterial genera whose abundances in the GM significantly differed between the alcoholic and non-alcoholic groups (see also Supplementary Fig. S3 for the genus-level analyses of the overall GM structures). The relative GM abundances of some dominant obligate anaerobes (*Bacteroides*, *Eubacterium*, and *Anaerostipes*) were significantly lower in the alcoholic patients than in the non-alcoholic group (*P*-value < 0.05; see Fig. 2(a)). In contrast, *Streptococcus* was characteristically higher in the GM of the alcoholic patients than in the GM of non-alcoholics (*P*-value < 0.05). The total abundance of other minor bacterial genera (Supplementary Fig. S3, termed *others*) was increased in the GM of alcoholics, wherein the relative abundance of each genus was below 1%, although the enrichment of each minor genus was not statistically significant. The minor bacteria enriched in the GM of alcoholic patients belonged to the families *Lactobacillaceae*, *Enterobacteriaceae,* and *Enterococcaceae* (Supplementary Fig. S4).

### Searching for other background factors relating to structural alteration of GM in alcoholics

**Figure 3:**
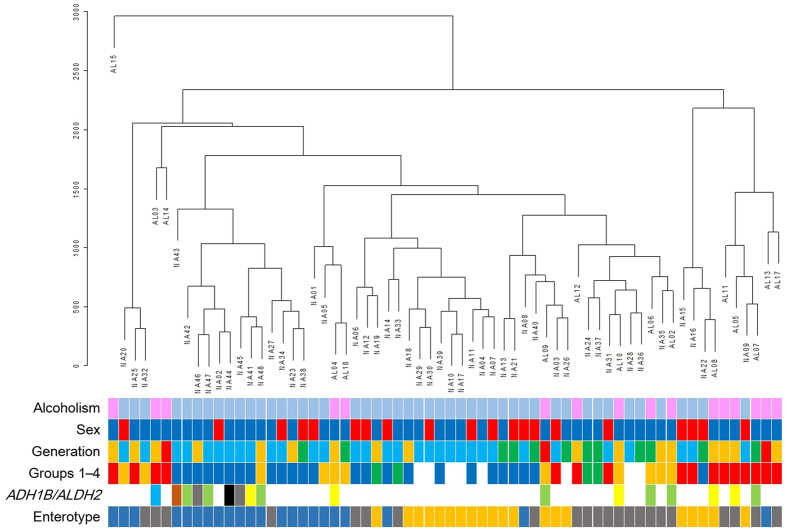
Hierarchical clustering of GM structures and their relationships to some background factors (alcoholism, sex, generation, drinking and smoking habits, the polymorphisms of *ADH1B* and *ALDH2*, and enterotypes). For alcoholism, *magenta* and *light-blue rectangles* indicate alcoholics and non-alcoholics, respectively. For sex, *deep-blue* and *red rectangles* indicate male and female, respectively. For generation, *sky-blue*, *green*, *orange*, and *red rectangles* indicate young (15–30 years old), mature (31–44 years old), middle-aged (45–65 years old), and elderly (66 years old or older), respectively. For drinking and smoking habits, *deep-blue*, *green*, *orange*, and *red rectangles* indicate Groups 1, 2, 3, and 4, respectively (see text for the group definitions). *White* represents the non-alcoholic volunteers who did not respond to the questionnaire. For the polymorphisms of *ADH1B* and *ALDH2*, *coloured rectangles* indicate the following genotypes: *blue*, *ADH1B* *1/*1 *ALDH2* *1/*1; *light green*, *ADH1B* *1/*2 *ALDH2* *1/*1; *brown*, *ADH1B* *1/*2 *ALDH2* *1/*2; *yellow*, *ADH1B* *2/*2 *ALDH2* *1/*1; *grey*, *ADH1B* *2/*2 *ALDH2* *1/*2; and *black*, *ADH1B* *2/*2 *ALDH2* *2/*2. For possible enterotypes of subjects, *blue*, *orange*, and *grey rectangles* indicate types 1, 2, and 3, respectively.

Next, we performed a hierarchical clustering analysis of the GM structures of all subjects (i.e., the alcoholic plus non-alcoholic subjects; see Fig. 3). The analysis suggests that the anomalous GM structures of alcoholics arises not only from alcoholism but also by other background factor(s) associated with alcoholics. Among the possible contributing factors, we can rule out age, enterotypes^25^ (Supplementary Fig. S5), and polymorphisms of alcohol dehydrogenase 1B [*ADH1B* (rs1229984)] and aldehyde dehydrogenase 2 [*ALDH2* (rs671)]^26^ (see Fig. 3, and the Discussion for further details). Notably, cigarette smoking has been associated with a moderately increased risk of CRC^27^ and many of our alcoholic subjects were habitual smokers (Supplementary Table S1). To investigate this issue, we re-classified the 56 questionnaire respondents (Supplementary Table S1; see also **Methods**) into four categories, based on their current drinking and smoking habits [Group 1, neither habitual drinker nor habitual smoker (*n* = 26); Group 2, habitual smoker but not habitual drinker (*n* = 4); Group 3, habitual drinker but not habitual smoker (*n* = 11); Group 4, habitual drinker and habitual smoker (*n* = 15) (for quantitative details of these definitions, see **Methods**)]. According to the hierarchical clustering analysis, the GM structures of alcoholics were clustered very similarly to the GM structures of Group 4 (*red rectangles*) and Group 3 (*orange rectangles*) (Fig. 3), suggesting that smoking habits are partly responsible for altering the GM structures in alcoholics.

**Figure 4:**
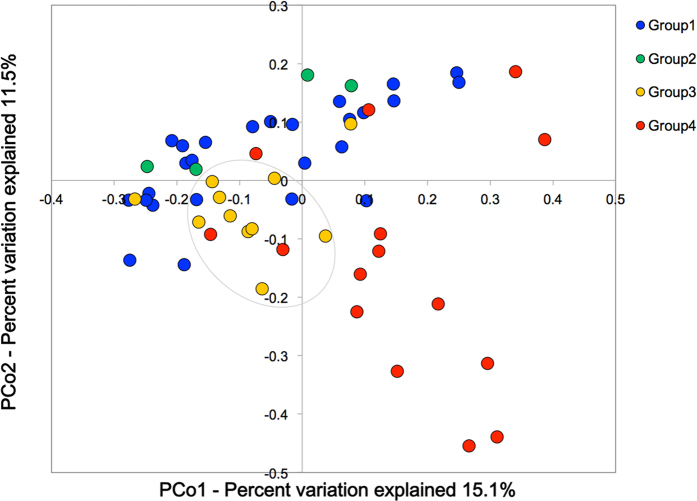
UniFrac PCoA plots of faecal bacterial communities of Groups 1–4 classified by their drinking and smoking habits. UniFrac PCoA plots. The *blue*, *green*, *yellow*, and *red* symbols denote Group 1 (*n* = 26), Group 2 (*n* = 4), Group 3 (*n* = 11, including 6 alcoholic patients), and Group 4 (*n* = 15, including 10 alcoholic patients), respectively. The *oval* indicates where the plots of Group 3 tend to segregate from those of non-drinkers (Groups 1 and 2).

Comparing Groups 1 and 3 and Groups 2 and 4 in an OTU analysis, we infer that habitual drinking alone enhances the inter-subject variations in the OTU numbers (Supplementary Fig. S6, Group 1 vs Group 3). The 56 questionnaire respondents were then subjected to a UniFrac PCoA analysis, and the distribution of their GM structures in the plots were grouped by their drinking and smoking habits (Groups 1–4 in Fig. 4). The GM structures of the subjects in Group 3 (*yellow symbol*s) appear to be segregated from those of non-drinkers (*i.e*., Groups 1 and 2; *blue* and *green symbols,* respectively). The GM structure was most diverse in Group 4 (*red symbols*). More quantitatively, the average UniFrac distance was significantly larger in Group 4 than in Groups 1, 2, and 3 (Supplementary Fig. S7); moreover, the UniFrac distances significantly differed between Groups 1 and 4, 2 and 4, and 3 and 4 (Supplementary Fig. S7). These results indicate that the β-diversity of the GM structures was much higher in Group 4 than in any other group.

**Figure 5:**
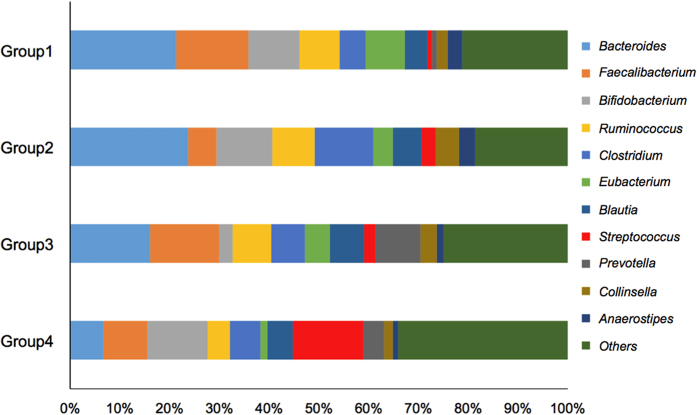
Relative abundances of bacterial genera in the faecal bacterial communities of Groups 1–4.

Figure 5 shows the relative abundances of the 10 most prominent bacterial genera in the GMs of these 4 groups. The GM of Group 4 was greatly enriched in *Streptococcus* (*P*-value < 0.05; see Supplementary Fig. S8C) relative to Group 1. *Streptococcus* was also higher in the GM of Group 4 than in those of Groups 2 and 3 (Fig. 5), although the enrichment was not statistically significant. The GM was poorer in some dominant obligate anaerobes in Group 3 than in Group 1 [*Bacteroides*, Bifidobacterium*, *Ruminococcus*, *Eubacterium*, and Anaerostipes*, where the asterisk denotes statistical significance (*P*-values < 0.05; see Supplementary Fig. S8B). Similarly, the GM of Group 4 was depleted in Bacteroides*, *Faecalibacterium*, Ruminococcus*, Eubacterium*, *Collinsela*, and Anaerostipes*, relative to Group 1 (see Supplementary Fig. S8C).

### Faecal phenotypic changes related to aerobic ethanol metabolism

**Figure 6:**
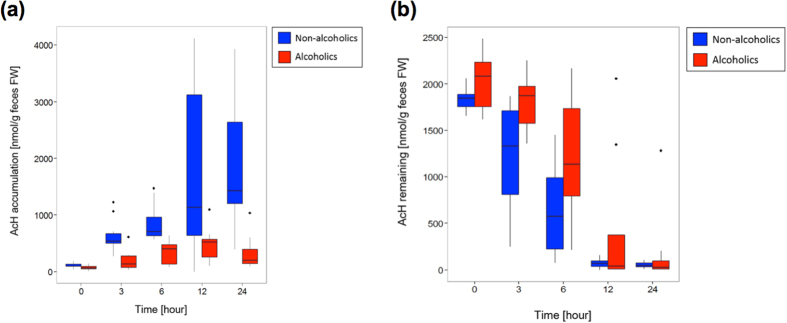
Aerobic faecal AcH metabolism. (**a**) Box-whisker plots comparing the courses of faecal AcH production between the non-alcoholic volunteers (blue, *n* = 10) and the alcoholic patients (red, *n* = 7). AcH accumulation was acquired during aerobic incubation with 22 mM ethanol at pH 7.0 and 37 °C. (**b**) Box-whisker plots comparing the initial rates of faecal AcH decomposition between the non-alcoholic volunteers (blue, *n* = 10) and the alcoholic patients (red, *n* = 7). Remaining AcH was determined during aerobic incubation with 175 ± 30 μM AcH (initial concentration) at pH 7.0 and 37 °C. We emphasise that in these assays, the faecal samples included both hard and soft types of formed faeces in each group, and the water contents were not determined. Thus, these data are not corrected for water contents. However, the reported average water contents of formed faeces do not significantly differ (68 ± 0.9% and 74 ± 0.3% for hard and soft forms, respectively)^58^.

Jokelainen *et al.*^8^ showed that human colonic contents aerobically incubated with 22 mM ethanol generate high levels of AcH. This study was pivotal in linking the aerobic ethanol metabolism of gut bacteria to ER-CRC pathogenesis^2^,^4^,^28^,^29^. More recently, we identified *Ruminococcus* and several other obligate anaerobes (*Collinsella*, *Prevotella*, *Coriobacterium*, and *Bifidobacterium*) as important potential AcH accumulators in the colon and rectum^17^. Because the GM structures were significantly altered in the alcoholic patients, we questioned whether the faecal phenotype (*i.e*., the faecal activity of ethanol oxidation under aerobic conditions) differs between alcoholics and non-alcoholics. To this end, we added 22 mM ethanol (pH 7.0; 37 °C) to the faecal samples of non-alcoholic volunteers and seven alcoholic patients (AL11–AL15, AL17, and AL18; Supplementary Table S1), and compared the courses of AcH production in the two groups. These assays were performed only on formed faeces (see Methods section and Fig. 6 for further details), and the initial ethanol concentration was chosen to approximate the typical ethanol concentration in the colon after a normal bout of alcohol consumption^30^,^31^,^32^,^33^. The faeces of the non-alcoholic subjects produced significant amounts of AcH (Fig. 6(a)). Jokelainen *et al.*^8^ reported similar results, despite the different assay conditions in their report^8^. By striking contrast, the faeces of the alcoholic patients produced no appreciable AcH (Fig. 6(a)), indicating significantly less AcH production from ethanol in the alcoholic group. Similar observations were made in the faecal samples of alcoholic patients after 2 weeks of abstinence (Supplementary Fig. S9). We also examined the faecal decomposition of AcH (170 ± 35 μM) under aerobic conditions. During the incubation, the AcH was slowly decomposed in the faecal samples of non-alcoholics, but more slowly decomposed in the samples of alcoholic patients (Fig. 6(b)). Thus, the poor ability of the alcoholics’ faecal samples to produce AcH from ethanol cannot be attributed to a higher faecal AcH decomposition rate than AcH production rate.

## Discussion

In the UniFrac analysis results (Fig. 1(b,c)), the microbial communities of alcoholics exhibited characteristically disordered patterns with higher β-diversity than those of non-alcoholic individuals. At the phylum level, the relative abundance of *Bacteroidetes* was significantly decreased in the GM of the alcoholic patients while *Proteobacteria* were apparently enriched, though the enrichment was statistically insignificant (Supplementary Fig. S2). Mutlu *et al.*^21^ reported a similar reduction of *Bacteroidetes* and enrichment of *Proteobacteria* in the colonic mucosa-associated microbiota of a subset of alcoholics. Moreover, using a mouse model, Bull-Otterson *et al.*^22^ reported that chronic ethanol consumption lowers the abundances of *Bacteroidetes* and *Firmicutes* and enhances *Proteobacteria* and *Actinobacteria*. These results suggest that chronic ethanol consumption generally diminishes the *Bacteroidetes* and enriches the *Proteobacteria* in the GM, regardless of human race and even animal species.

The GM structures of the alcoholic patients and non-alcoholic volunteers were further compared at the genus level. The GM of alcoholic patients were significantly depleted of the three dominant genera in the guts of non-alcoholics, namely, *Bacteroides*, *Eubacterium*, and *Anaerostipes*, (Fig. 2(a)), and were also diminished in other prominent bacteria (i.e., *Bifidobacterium* and *Ruminococcus*; see Supplementary Fig. S3), although the diminution was not statistically significant. By contrast, the relative abundances of *Streptococcus* and *Coprobacillus*, which are minor in the guts of non-alcoholics, were significantly increased in the GM of alcoholics (*P* < 0.05) (Fig. 2(a)). The total abundance of other minor bacterial families (whose genus members comprise below 1% of the GM in non-alcoholic participants) was also increased in the GM of alcoholic patients, although the increase was not statistically significant (Supplementary Fig. S4). However, the OTU numbers in the GM of alcoholic patients were not appreciably increased (Fig. 1(a)). Thus, the diminution of prominent bacteria, along with the enrichment of *Streptococcus* and other minor bacterial species, might largely explain the high β-diversity observed in the GM structures of the alcoholic patients (see above).

According to the hierarchical clustering analysis of the GM structures (Fig. 3), habitual drinking and smoking probably caused the deviation of GM structures among the investigated subjects. The synergistic effect of habitual drinking and smoking on the structural alteration of GM was also supported by the UniFrac PCoA analysis (Fig. 4). Specifically, habitual drinking alone could segregate the GM structures of drinkers and non-drinkers (enclosed by *ovals* in Fig. 4), whereas habitual smoking alone appeared to have no such effect (Fig. 4, *green symbols*). However, combined habitual drinking and smoking led to apparent dysbiosis of the GM structures (Fig. 4, *red symbols*; see also Supplementary Fig. S7). The phylogenetics indicated that habitual drinking alone enriches *Streptococcus* and other minor gut bacteria while reducing *Bacteroides* and *Ruminococcus* (Group 1 vs. Group 3 in Fig. 5), and that habitual smoking exacerbates this trend (Group 3 vs. Group 4 in Fig. 5; see also Supplementary Fig. S8).

It should be noted that hierarchical clustering analysis predicted no appreciable association of the anomalous GM structures of alcoholics with age, enterotypes^25^, and the genetic polymorphisms of *ADH1B* (rs1229984) and *ALDH2* (rs671)^26^ (see Fig. 3). Specifically, some of the middle-aged and elderly people were apparently co-clustered with young and mature participants, while others were distinct from these groups (Fig. 3). Because alcoholism and age are associated, and many of our alcoholic subjects were middle-aged or elderly, the alcoholics are expected to cluster among the older groups. As individuals age, their preferences and lifestyles, including their dietary, smoking, and drinking habits, also tend to change^34^. Unlike age alone, these changes should be reflected in the subjects’ GM structures (see Results). Genetic polymorphisms of *ADH1B* and *ALDH2* can potentially influence the drinking behaviours of subjects who have begun drinking^35^, and could be related to alcoholism susceptibility in Japanese individuals^36^. All nine of the alcoholic subjects in the present study exhibited a *1/*1 *ALDH2* genotype, and eight of them exhibited *ADH1B* genotypes of *1/*2 (4 subjects) or *2/*2 (4 subjects) (see Supplementary Table S1). However, the hierarchical clustering analysis showed that subjects possessing these genotypic combinations (*green* and *yellow rectangles* shown in Fig. 3) were almost indistinguishably distributed among both alcoholics and non-alcoholic subjects. These results suggest that the *ADH1B* and *ALDH2* genotypes alone do not primarily affect the GM structures, although they are potential indicators of later drinking habits.

The mechanism of the observed alcoholism-induced alteration of GM structures might be complex and related to multiple pathophysiological factors besides alcoholism (chronic ethanol consumption) itself. The continuous presence of ethanol in the colon and rectum of alcoholics could be at least partially responsible for the altered GM structures. Specifically, a normal bout of alcohol consumption raises the blood ethanol concentration to an estimated 22 mM^30^,^32^,^33^. Chronic ethanol consumers retain ethanol in their blood for prolonged periods^32^,^33^. Because of its high water solubility, the ethanol levels inside the colon and blood are equal while the ethanol persists in the human body^30^. Previously, we isolated and examined more than 500 bacterial strains from the faeces of alcoholics^17^, and observed no adverse effects of ethanol (22 mM) on the growth of specific intestinal bacteria. Instead, the observed alteration of the GM structure in alcoholics appears to be related to the oxygen tolerance of gut bacteria. Specifically, the guts of alcoholics were significantly depleted of dominant obligate anaerobes (see above), and enriched in aerotolerant (facultative anaerobic) groups such as *Streptococcus* and other minor bacterial species in the families *Lactobacillaceae*, *Enterobacteriaceae*, and *Enterococcaceae* (see Supplementary Fig. S4). Given that ethanol induces ROS production through various cellular processes^5^,^6^, the altered GM structure in alcoholics might be related (at least partly) to the tolerance of gut bacteria to ethanol-induced ROS. Within the colorectal environment, ethanol could induce ROS formation through various mechanisms^5^,^6^; indeed, gut mucosal cells have been shown to mediate ROS formation via oxidative metabolism of ethanol^37^. Although most of the ROS produced in this way could be eliminated by ROS-scavenging systems in the colon and rectum, a small fraction could incidentally remain and diffuse into the colorectal contents, with numerous deleterious consequences (*e.g*., oxidative stresses) on the survival of gut bacteria. Thus, faecal bacteria inhabiting the colorectal environment of alcoholics likely face higher ROS exposure than those inhabiting non-alcoholic subjects. Facultative anaerobes are more tolerant to oxygen and ROS than obligate anaerobes, which cannot use oxygen as a terminal electron acceptor, and so are killed by both factors^38^. This mechanism can reasonably account, at least partly, for the reduced abundance of obligate anaerobes and the enrichment of aerotolerant *Streptococcus* in the GM of the alcoholic patients. Further supporting this mechanism, the phylum *Proteobacteria* is generally enriched in the GM of the alcoholic patients (as described above; see also Mutlu *et al.*^21^ and Bull-Otterson *et al.*^22^) because most of the gut bacteria belonging to this phylum are facultative anaerobes^38^.

The proposed ROS-mediated alteration of GM structures also explains the exacerbation of GM alteration by habitual smoking. Cigarette smoke contains numerous chemicals that mediate ROS formation and oxidative stress^39^. Thus, the habitual ingestion of alcoholic beverages probably increases the efficiency of delivery of substances in cigarette smoke to the large intestine, further promoting ROS-mediated alteration of the GM structures in chronic ethanol consumers. Supporting this hypothesis, habitual smoking increases the risk of CRC in heavy drinkers^26^ and alcoholics^3^. Habitual smoking and/or habitual drinking affects the relative abundance of faecal bacteria to varying extent; some species are probably more susceptible to substances in cigarette smoke than others (Fig. 5, see also Supplementary Fig. S8). Interestingly in this context, tar-resistant (or tarphilic) *Streptococcus* species^40^ are enriched in the disordered microbial communities inhabiting the saliva and upper respiratory tracts of cigarette smokers^41^,^42^; notably, *Streptococcus* were highly enriched in the disordered GM of alcoholics with smoking habits (see Fig. 5, Group 4).

It must be stressed that other factors, such as nutrition, will also affect the GM structure of alcoholic patients. Many alcoholics are malnourished because they ingest few essential nutrients and because ethanol and its metabolism prevent the body from properly absorbing, digesting, and using those nutrients^43^. The impact of malnutrition on the microbiota of the murine small intestine was recently reported^44^. The effect of malnutrition on the altered GM structures of alcoholics should be investigated in future studies.

According to the *in vitro* faecal assay results, the faecal samples of non-alcoholics produced appreciable levels of AcH from ethanol under aerobic conditions (see Fig. 6(a)). Similar observations were reported by Jokelainen *et al.*^8^, who investigated ethanol metabolism by faecal aerobes under different assay conditions from the present study. However, alcoholic faecal samples produced no appreciable AcH from ethanol under the same conditions in the present study (Fig. 6(a)). This faecal phenotype of alcoholics was unchanged after 2 weeks of abstinence (Supplementary Fig. S9). Given that human GM are probably long-term stable^19^, the diminished capacity of ethanol oxidation in the faeces of our alcoholic subjects was most likely related to the altered GM structure, rather than the temporary alteration of the bacterial transcriptome structures (*i.e*., metabolic states). Having characterised the GM structures of the alcoholic patients, the three prominent faecal bacteria in both the alcoholic and non-alcoholic groups were *Bacteroides*, *Bifidobacterium,* and *Ruminococcus* (see Fig. 5). We recently showed that *Bacteroides* were virtually inert in aerobic ethanol metabolism, whereas some members of *Bifidobacterium*, *Ruminococcus*, *Collinsella*, and *Prevotella* are potential AcH accumulators^17^. *Ruminococcus* species particularly dominate the potential AcH accumulators and are thought to play important roles in AcH production through aerobic ethanol metabolism in the colon and rectum^17^. Thus, we can consistently attribute the reduced ethanol oxidation ability of the alcoholic faecal samples to the decreased numbers of *Ruminococcus* (and other potential AcH accumulators such as *Bifidobacterium*, *Collinsella*, and *Prevotella*)^17^ in the GM of these patients (Fig. 2(b)). In this context, a culture-based analysis revealed that oral administration of ciprofloxacin decreases the rate of ethanol elimination in humans^16^, probably because this drug reduces the proportions of aerobes and facultative anaerobes in the human intestine^16^. However, in recent culture-free GM analysis, ciprofloxacin administration to human subjects (outpatients with urinary tract infections) decreased the relative abundances of various obligate anaerobes, including *Bifidobacterium* and *Ruminococcus*^45^. Thus, ciprofloxacin adversely affects not only aerobes and facultative anaerobes, but also obligate anaerobes. These observations corroborate the proposed importance of *Bifidobacterium*, *Ruminococcus* and other anaerobic AcH accumulators in faecal ethanol metabolism under aerobic conditions^17^.

It has been proposed that the mechanisms of ER-CRC are closely related to ethanol metabolism, which mediates the formation of two important cancer-causing agents, ROS and AcH^2^. The present finding that the dominant obligate anaerobes are diminished in the GM of alcoholics (see Fig. 2) is consistent with the ethanol-induced formation of ROS in the colorectal environment^5^,^6^,^37^ and might also be related to the ROS-mediated pathogenesis of CRC^2^,^7^. In proposing the AcH-mediated pathogenesis of ER-CRC, we emphasise that the very poor ethanol oxidising ability observed in the faeces of alcoholics does not necessarily contradict the proposed role of AcH as a risk factor in ER-CRC^2^,^28^,^29^. The observed faecal phenotype is of little physiological significance because the colorectal environment is strictly anaerobic, whereas in the present study, the faecal ethanol metabolism was assayed under aerobic conditions. Given that CRC develops from mucosal cells, the expected main players in ER-CRC pathogenesis are populations (biofilms or microcolonies) of AcH-accumulating bacteria inhabiting the colorectal mucosal surface, rather than inhabitants of the strictly anaerobic interior luminal^14^,^15^. Because the colorectal mucosal surface is usually aerobic^46^ and colorectal mucosal cells mediate ROS formation through oxidative ethanol metabolism^5^,^6^,^37^, bacterial cells inhabiting the colorectal mucosal surface should be exposed to higher and more sustained concentrations of O_2_ and ROS after drinking than those inhabiting the strictly anaerobic regions inside the colorectal contents. Recently, we reported that ethanol oxidation by *Ruminococcus* and some other anaerobic potential AcH accumulators is mediated by O_2_ and/or ROS^17^, and that AcH accumulates to levels exceeding the MMC (50 μM). Similarly, some intestinal aerobes and facultative anaerobes, which are likely enriched near the aerobic colorectal mucosal surface, could also accumulate AcH^14^,^15^,^16^ under exposure to O_2_ and ROS. For example, many aerobes and facultative anaerobes show catalase activity^47^, which removes H_2_O_2_ in response to oxidative stress. Because this enzyme also catalyses the oxidation of ethanol to AcH^47^, catalase-positive bacteria could potentially accumulate AcH in the presence of ethanol and ROS. These potential AcH accumulators might also accumulate AcH to high levels *in vivo*, particularly when forming biofilms on the mucosal surface^17^. In these biofilms, the local cell densities can exceed 1.0 × 10^9^ cells/cm^3 ^48,49. Biofilms of bacteria related to potential AcH accumulators on the colorectal mucosa have been confirmed by different methods^21^,^48^,^49^,^50^,^51^,^52^. Thus, biofilms of such bacterial species on the colorectal mucosal surface likely mediate the ‘microscopic’ local production of AcH from ethanol in an O_2_- and/or ROS-dependent manner. Exposure to super-MMC levels of AcH over prolonged periods predisposes the guts of alcoholics to carcinogenesis^17^. Thus, if bacterially produced AcH in the colon and rectum is implicated in ER-CRC pathogenesis, prolonged exposure of the aerobic colorectal mucosa to ‘microscopic’ local AcH should be of mechanistic importance^14^,^15^, whereas ‘macroscopic’ AcH levels in the anaerobic lumen may play a negligible role.

Finally, components of human GM have been implicated in CRC pathogenesis. These components impart genotoxic stresses to the intestinal epithelial cells, promoting genetic and epigenetic alterations of the epithelial cells, eventually leading to CRC^53^,^54^. However, whether (or how) the alcoholic GM structure primarily contributes to CRC development remains unclear, because the changes leading to CRC occur over many years, even decades. In this context, the absence of colonoscopy or biopsy data from the cohort of alcoholics and non-alcoholics would be a limitation of this study. Longitudinal follow-up studies of GM structures using alcoholic patients with colorectal neoplasia and cancer-free individuals would be needed to address this issue. Moreover, in future study, we should clarify the microbiota structures of the colorectal mucosal biofilms of ER-CRC patients to further understand the proposed role of the colorectal mucosa-associated microbes in the pathogenesis of ER-CRC.

## Methods

### Subjects and faecal sample collection

The sixteen alcoholic patients participating in this study (Supplementary Table S1) were Japanese men making their first visit to the Kurihama Medical and Addiction Center for alcoholism treatment. All study subjects met the following criteria: (i) the DSM-IV criteria for alcohol dependence; (ii) continued drinking until at least 7 days before the faecal sample collection; (iii) had never used alcohol-aversive drugs; (iv) no suspicion of liver cirrhosis; and (v) no antibiotic treatment.

The 48 non-alcoholic subjects were healthy volunteers (all Japanese) recruited from the Graduate School of Engineering, Tohoku University, and the School of Veterinary Medicine, Azabu University. Their mean (±S.D.) age was 29 ± 11 years (range 19–59 years), and their mean (±S.D.) BMI was 21.9 ± 3.4. These subjects had no health problems, took no drugs, and underwent no antibiotic treatment. All subjects were informed of the purpose of the study.

This study was carried out according to the ethical principles of the Declaration of Helsinki. Analysis of genetic polymorphisms of *ADH1B* and *ALDH2* was carried out according to the Ethics Guidelines for Human Genome/Gene Analysis Research by the Ministry of Education, Culture, Sports, Science and Technology; Ministry of Health, Labour and Welfare; and Ministry of Economy, Trade and Industry of Japan. The Ethics Committees of the Kurihama Medical and Addiction Center; the School of Veterinary Medicine, Azabu University; and the Graduate School of Engineering, Tohoku University, reviewed and approved the proposed studies (G26, 038, and 10B-2/13A-2, respectively), and all participants gave written informed consent to participate in the study. Each subject received a questionnaire concerning his or her drinking and smoking history. Among the 64 eligible volunteers, 56 responded to the questionnaire.

To study the effects of drinking and smoking habits on the GM structure, we scored the volunteer’s drinking and smoking habits on four-point scales. Drinking habits were scored as: 1) do not drink; 2) drink approximately once per week; 3) drink approximately three times per week; 4) drink every day. Smoking habits were scored as: 1) non-smoker; 2) smoke, but not every day; 3) smoke every day, but no more than one pack of cigarettes per day, 4) smoke one pack of cigarettes or more every day. Among the alcoholic patients, there were four ex-smokers. Considering their long-term cessation of smoking (22, 24, 15 and 2 years), these subjects were assigned a smoking habit score of 1. Based on their scores, all subjects who submitted to the GM structure analysis (the 16 alcoholic patients and 40 non-alcoholic volunteers who responded to the questionnaire) were classified into one of four groups (Groups 1 through 4; Supplementary Table S1): Group 1, drinking and smoking scores of 1 or 2 (i.e., neither habitual drinker nor habitual smoker; *n* = 26); Group 2, drinking score of 1 or 2 but smoking score of 3 or 4 (i.e., habitual smoker but not habitual drinker; *n* = 4); Group 3, drinking score of 3 or 4 but smoking score of 1 or 2 (habitual drinker but not habitual smoker; *n* = 11, including 6 alcoholic patients), and Group 4, drinking and smoking scores of 3 or 4 (i.e., habitual drinker and habitual smoker; *n* = 15, including 10 alcoholic patients).

Immediately after collection, the faecal samples were transferred to an anaerobic gas-producing pouch (AnaeroPack-Kenki, Mitsubishi Gas Chemical Co., Tokyo Japan), and were frozen on dry ice at −79 °C until required for analysis. Diarrheal faeces were not collected.

### Genotyping of *ADH1B* and *ALDH2*

The subjects’ dried saliva samples were directly genotyped without DNA extraction by the TaqMan assay, as described previously^55^.

### DNA isolation from faeces

Bacterial genomic DNA was isolated from the faecal samples and purified as described by Morita *et al.*^56^ with minor modifications. Typically, 3 g (wet weight) of a faecal sample was suspended in an appropriate volume of phosphate-buffered saline (PBS; pH 7.0). Food debris and other non-microbial contaminants in the suspension were removed by passing through a 100-μm filter. The filtrate was centrifuged at 5,000 rpm for 10 min. The precipitate was suspended in 10 ml of 10 mM Tris-HCl/1 mM EDTA (TE) and incubated with 15 mg/ml lysozyme (final concentration, Sigma–Aldrich, St. Louis, MO, USA) at 37 °C for 1 h. Purified achromopeptidase (Wako Pure Chemical Industries, Tokyo, Japan) was added at a final concentration of 2,000 units/ml. After further incubation at 37 °C for 30 min, sodium dodecyl sulfate and proteinase K (Merck, Darmstadt, Germany) were added to the suspension at final concentrations of 1% (w/v) and 1 mg/ml, respectively, and the mixture was incubated at 55 °C for 1 h. The resultant cell lysate was treated with phenol/chloroform/isoamyl alcohol (Life Technologies Japan, Tokyo, Japan). DNA was precipitated by adding ethanol followed by centrifugation at 3,300 × *g* at 4 °C for 15 min. The DNA precipitate was washed with 75% ethanol, dried, and dissolved in TE. DNA samples were treated with 1 mg/ml (final concentration) RNase A, (Wako Pure Chemical Industries) at 37 °C for 30 min and precipitated by adding an equal volume of a mixture of 20% polyethylene glycol 6000 and 2.5 M NaCl. DNA was precipitated by centrifugation at 8,000 × *g* at 4 °C, washed with 75% ethanol, and dissolved in TE.

### 454 pyrosequencing of bacterial 16S rRNA gene amplicons

The V1–V2 region of the 16S rRNA gene (16S) was amplified using a forward 27Fmod-454A primer (5′-CCATCTCATCCCTGCGTGTCTCCGACTCAGNNNNNNNNNNagrgtttgatymtggctcag)^57^ and a reverse 338R-454B primer (CCTATCCCCTGTGTGCCTTGGCAGTCTCAGtgctgcctcccgtaggagt). The uppercase letters in the 27Fmod primer comprise the nucleotide sequence of 454 primer A; the series of Ns is a 10-base barcode sequence that is unique to each sample; and the lowercase letters indicate the 27Fmod primer sequence. The replacement of the conventional 27F primer with the 27Fmod primer enabled a quantitative and accurate analysis of the GM structures^57^. In the nucleotide sequence of the 338R-454B primer, the uppercase and lowercase letters comprise the sequences of 454 primer B and the 338R primer, respectively.

The polymerase chain reaction (PCR) was completed in 1× Ex Taq PCR buffer (50 μl) containing dNTP (2.5 mM), Ex Taq polymerase (Takara Bio, Shiga, Japan), both primers (10 μM), and 40 ng of the isolated DNA, using a 9700 PCR system (Life Technologies Japan). The thermal cycling conditions were 96 °C for 2 min, followed by 20 cycles of 96 °C for 30 s, 55 °C for 45 s, and 72 °C for 1 min, and a final extension of 72 °C for 10 min. The PCR products (approximately 370 bp) were confirmed by agarose gel electrophoresis, purified by AMPure XP magnetic purification beads (Beckman Coulter, Brea, CA, USA), and quantified using a Quant-iT PicoGreen dsDNA Assay Kit (Life Technologies, Japan). Approximately equal amounts of PCR amplicons from each sample were combined and pyrosequenced using either a 454 GS FLX Titanium instrument or a 454 GS JUNIOR instrument (Roche Applied Science, Penzberg, Upper Bavaria, Germany), according to the manufacturer’s instructions.

### Processing of raw sequence data

Raw sequence data (16S reads) were filtered and de-noised as described previously^57^. Briefly, 16S reads were assigned to each sample based on its barcode sequence information. 16S reads with no PCR primer sequences at both termini, and those with an average quality value below 25, were removed. The 16S reads containing possible chimeric sequences, with Basic Local Alignment Search Tool (BLAST) match lengths (against reference sequences in the database) below 90%, were also removed. Finally, the filter-passed reads were stripped of their primer sequences for further analysis.

### Data analyses

Three thousand filter-passed 16S reads per sample were used for OTU and UniFrac distance analyses^23^. In the OTU analysis, the 16S reads were clustered using the UCLUST program (www.drive5.com) with a pair-wise identity cutoff of 96%. Representative sequences of each OTU were assigned to bacterial species using BLAST. The complete genome database for the BLAST assignments was constructed by collecting genome sequences from the NCBI FTP site (ftp://ftp.ncbi.nih.gov/genbank/, December 2011) and the Hattori laboratory in-house database. Sequence similarity thresholds of 70% and 97% were applied to the phylum and genus assignments, respectively. The OTU numbers were estimated by extrapolation (Chao1 and ACE) using the vegan package (v2.0-5) for R (v2.15.2). From the UniFrac distance analysis, we determined the dissimilarity (distance) between two communities in the phylogenetic tree, based on the fraction of branch length shared by both communities.

The hierarchical clustering of GM structures of alcoholics and non-alcoholics was performed based on relative genus abundance by means of a complete linkage hierarchical clustering technique using R.

For enterotyping, samples were clustered based on relative genus abundances using the Jensen-Shannon divergence distance and the Partitioning-Around-Methods (PAM) clustering algorithm^25^.

### Faecal ethanol metabolism

In the faecal AcH production and decomposition assays, the initial substrate concentrations were chosen to approximate their typical values in the colon after a normal bout of alcohol consumption^30^,^31^,^32^,^33^. Formed faeces were collected from alcoholic patients (AL11–AL15, AL17, and AL18) and non-alcoholic subjects. To homogenise the storage conditions of the faecal samples, all faecal samples were immediately frozen in dry ice after collection, maintained at −78 °C for 1 day, then thawed at room temperature prior to the assays. A pilot study confirmed that fresh and frozen faecal samples yielded the same results. Typically, a faecal sample (6 g fresh weight) was suspended in 30 ml of PBS. For AcH production assay, 4.0 ml of this faecal suspension was mixed with 4.0 ml of 44 mM ethanol in PBS, and the mixture was subdivided into 1.2-ml aliquots. The aliquots were incubated at 37 °C for 1, 3, 6, 12, or 24 h and mixed with 100 μl of 6 M perchloric acid at specified times to stop the reaction. For AcH decomposition assay, the faecal suspension (4.0 ml) was mixed with an equal volume of 400 μM AcH in PBS, and treated as described for the AcH production assay. A blank containing faecal suspension mixed with an equal volume of PBS but without ethanol and AcH was also prepared. The AcH concentration in the resultant mixture was determined by head-space gas chromatography. In these assays, we obtained the average of duplicate experiments. To determine the faecal AcH production, we plotted the average AcH produced during the incubation (nmol AcH produced/g faeces fresh weight) as a function of time and compared the results of the alcoholic and non-alcoholic groups using box-and-whisker plots. The faecal AcH decomposition was analysed similarly, but using the average remaining AcH at the end of the incubation (nmol AcH remaining/g faeces fresh weight). We emphasise that faecal samples incubated at 100 °C for 30 min could neither oxidise ethanol nor decompose AcH. Therefore, any faecal AcH production and decomposition occurred by enzymatic or microbial processes alone.

## Additional Information

**Accession codes:** The filtered amplicon sequences were deposited in DDBJ/GenBank/EMBL, under the accession number DRA003809.

**How to cite this article**: Tsuruya, A. *et al.* Ecophysiological consequences of alcoholism on human gut microbiota: implications for ethanol-related pathogenesis of colon cancer. *Sci. Rep.* **6**, 27923; doi: 10.1038/srep27923 (2016).

## Supplementary Material

Supplementary Information
