# Supplementary material for: Ecophysiological consequences of alcoholism on human gut microbiota: implications for ethanol-related pathogenesis of colon cancer
Source: Sci Rep. 2016 Jun 13;6:27923. doi: 10.1038/srep27923 (PMC4904738; doi:10.1038/srep27923)
Supplement: Supplementary Information [file srep27923-s1.pdf]

SUPPLEMENTARY INFORMATION

TO

Ecophysiological consequences of alcoholism on human gut microbiota: implications for ethanol-related pathogenesis of colon cancer

BY

Atsuki Tsuruya, Akika Kuwahara, Yuta Saito, Haruhiko Yamaguchi, Takahisa Tsubo, Shogo Suga, Makoto Inai, Yuichi Aoki, Seiji Takahashi, Eri Tsutsumi, Yoshihide Suwa, Hidetoshi Morita, Kenji Kinoshita, Yukari Totsuka, Wataru Suda, Kenshiro Oshima, Masahira Hattori, Takeshi Mizukami, Akira Yokoyama, Takefumi Shimoyama, Toru Nakayama

---

Table of Contents

Supplementary materials	page
Supplementary Table S1 ··	2
Supplementary Figure S1 ··	4
Supplementary Figure S2 ··	5
Supplementary Figure S3 ··	6
Supplementary Figure S4 ··	7
Supplementary Figure S5 ··	8
Supplementary Figure S6 ··	11
Supplementary Figure S7 ··	12
Supplementary Figure S8 ··	13
Supplementary Figure S9 ··	14

Supplementary Table S1. Characteristics of the alcoholic and non-alcoholic subjects included in the GM analyses							
Sample ID	Age	Sex	Drinking habit score[*1]	Smoking habit score[*1]	Group[*2]	*ADH1B*	*ALDH2*
Alcoholics							
AL02	63	male	4	1	3	*1/*2	*1/*1
AL03	45	male	4	4	4	*1/*1	*1/*1
AL04	56	male	4	1	3	*2/*2	*1/*1
AL05	60	male	4	4	4	*2/*2	*1/*1
AL06	34	male	4	1	3	*1/*2	*1/*1
AL07	41	male	4	4	4	*1/*2	*1/*1
AL08	52	male	4	4	4	*2/*2	*1/*1
AL09	66	male	4	1	3	*1/*2	*1/*1
AL10	60	male	4	1	3	*2/*2	*1/*1
AL11	49	male	4	4	4		
AL12	52	male	4	3	4		
AL13	71	male	4	4	4		
AL14	68	male	4	4	4		
AL15	50	male	4	4	4		
AL17	64	male	4	4	4		
AL18	40	male	4	1	3		
Non-alcoholics[*3]							
NA01	21	female	2	1	1		
NA02	21	female	1	1	1		
NA03	22	female	1	1	1		
NA04	20	male					
NA05	20	male	3	1	3		
NA06	21	female	1	1	1		
NA07	21	female	1	1	1		
NA08	22	female	1	1	1		
NA09	21	female	3	3	4		
NA10	19	male					
NA11	20	female	1	1	1		
NA12	21	female	1	1	1		
NA13	33	male	1	1	1		
NA14	19	female	1	1	1		
NA15	19	female	3	3	4		
NA16	21	female	3	4	4		
NA17	19	male					
NA18	50	male	2	1	1		
NA19	50	male	2	3	2		
NA20	28	female	3	1	3		
NA21	39	female	1	1	1		

NA22	33	female	1	1	1		
NA23	45	male	2	1	1		
NA24	35	male	2	3	2		
NA25	50	male	3	3	4		
NA26	31	male		3			
NA27	29	male	2	1	1		
NA28	28	male		3			
NA29	28	male		3			
NA30	23	female		1			
NA31	22	female	3	3	4		
NA32	23	male	3	1	3		
NA33	22	male	2	3	2		
NA34	22	female	2	1	1		
NA35	49	male	3	1	3		
NA36	41	male		3			
NA37	37	male	2	3	2		
NA38	34	female	2	1	1		
NA39	26	male	2	1	1		
NA40	36	female	1	1	1		
NA41	26	male	1	1	1	*2/*2	*1/*1
NA42	25	male	2	1	1	*1/*2	*1/*1
NA43	23	male	1	1	1	*1/*2	*1/*2
NA44	30	male	1	1	1	*2/*2	*2/*2
NA45	24	male	2	1	1	*2/*2	*1/*2
NA46	59	male	1	1	1	*2/*2	*1/*2
NA47	23	male	2	1	1	*1/*2	*1/*1
NA48	56	male	4	1	3	*1/*2	*1/*1

[*1] Drinking-habit and smoking-habit scores are defined as described in the **Methods** section.

[*2] Groups are classified according to drinking and smoking habits. Group 1, neither habitual drinker nor habitual smoker; Group 2, habitual smoker but not habitual drinker; Group 3, habitual drinker but not habitual smoker; and, Group 4, habitual drinker and habitual smoker (see the **Methods** section for the quantitative details of the Group definitions)

[*3] The non-alcoholic volunteers without one or both of the drinking-habit and smoking-habit scores are those who did not respond to the questionnaire.

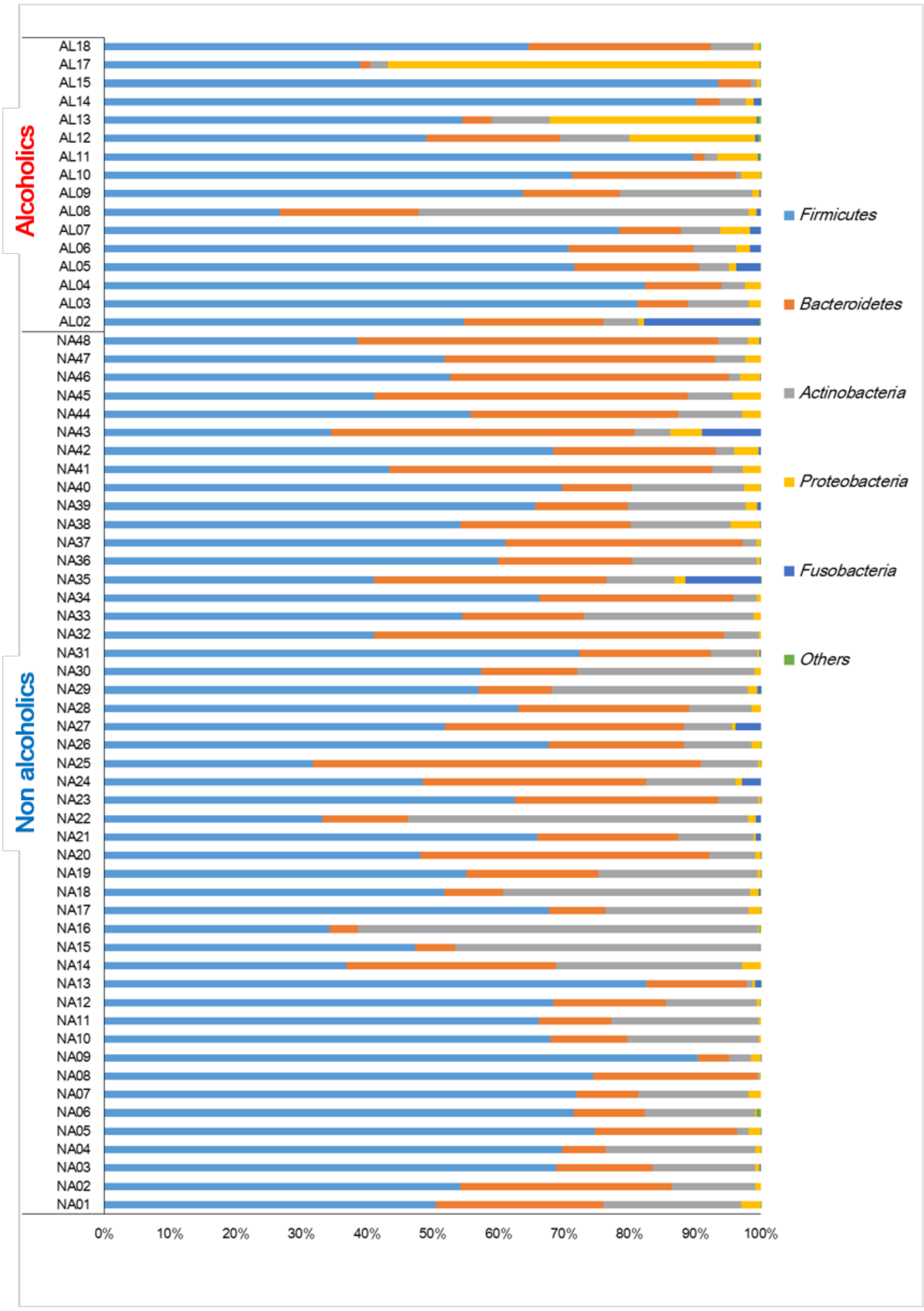

Supplementary Figure S1 | Relative abundances of bacterial phyla in the fecal bacterial communities of alcoholics (*upper*) and non-alcoholics (*lower*).

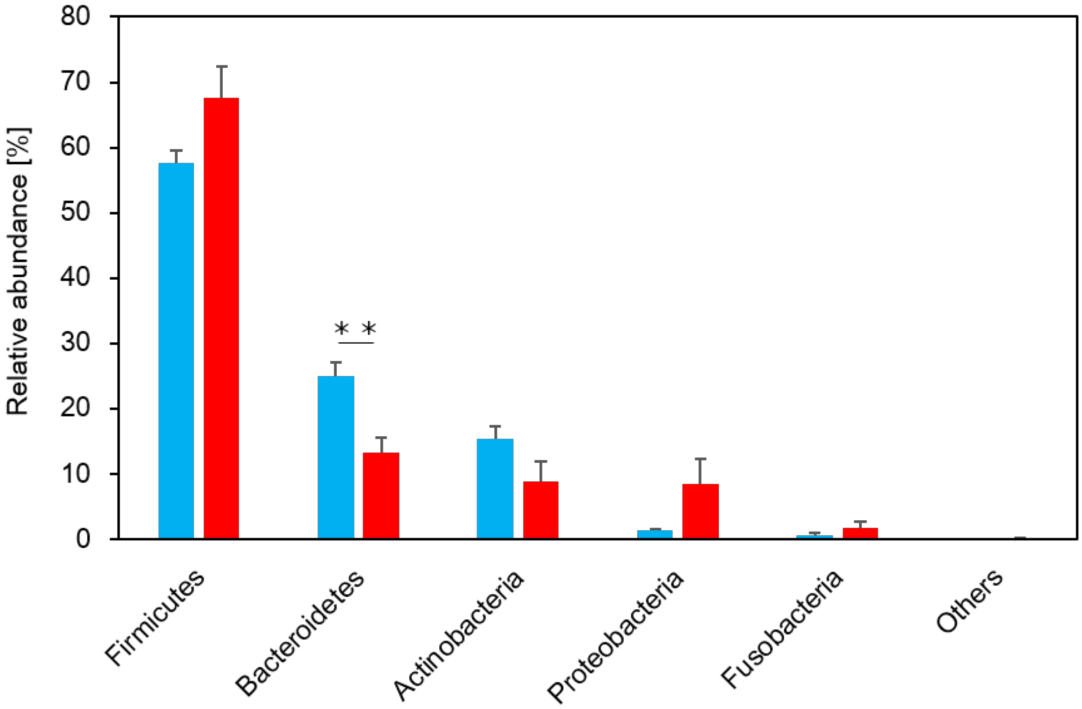

Supplementary Figure S2 | Comparison of relative abundances of five prominent bacterial phyla in the fecal bacterial communities between non-alcoholics (*blue bars*) and alcoholics (*red bars*). ** P-value < 0.01 (Welch'stest).

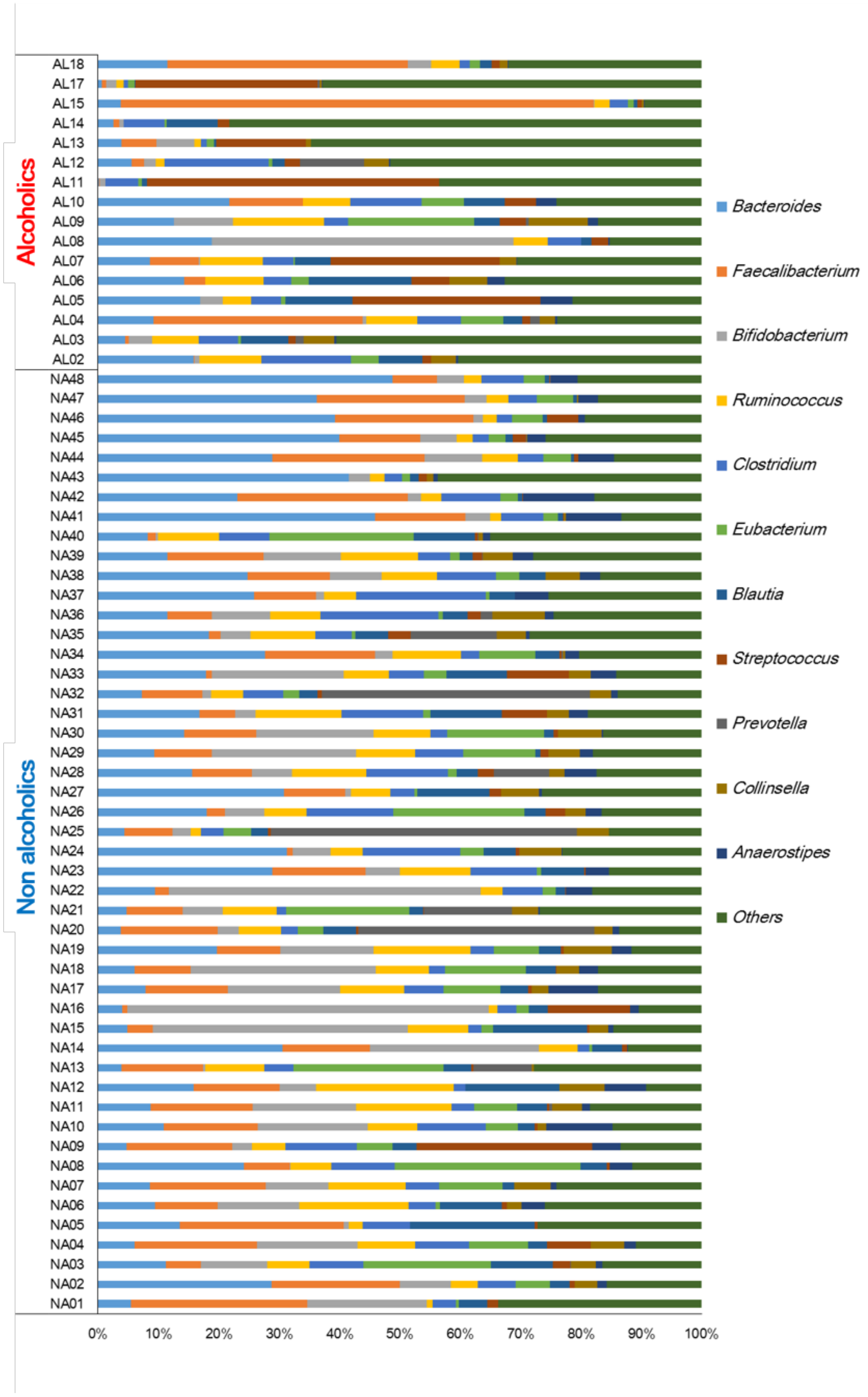

Supplementary Figure S3 | Overall GM structures of the alcoholic (AL) and non-alcoholic (NA) subjects analyzed at the genus level.

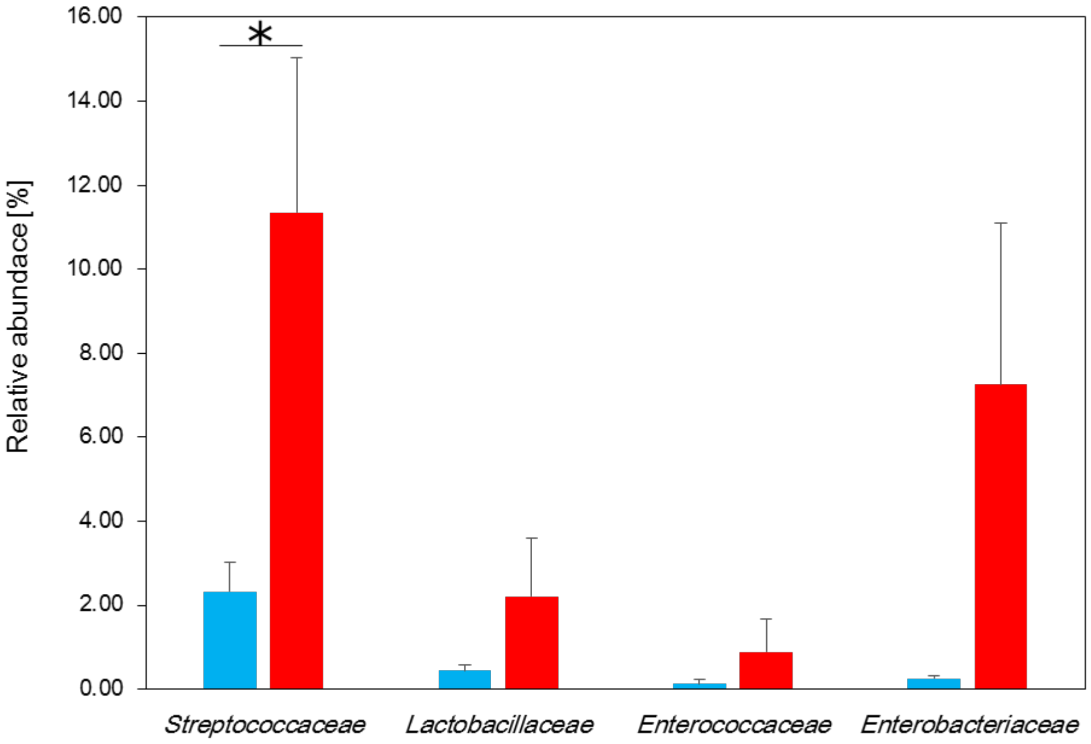

Supplementary Figure S4 | Relative abundances of the families *Streptococcaceae*, *Lactobacillaceae*, *Enterococcaceae,* and *Enterobacteriaceae* in the fecal bacterial communities were compared between non-alcoholics (*blue bars*) and alcoholics (*red bars*). * *P*-value < 0.05 (Welch'stest).

(A)

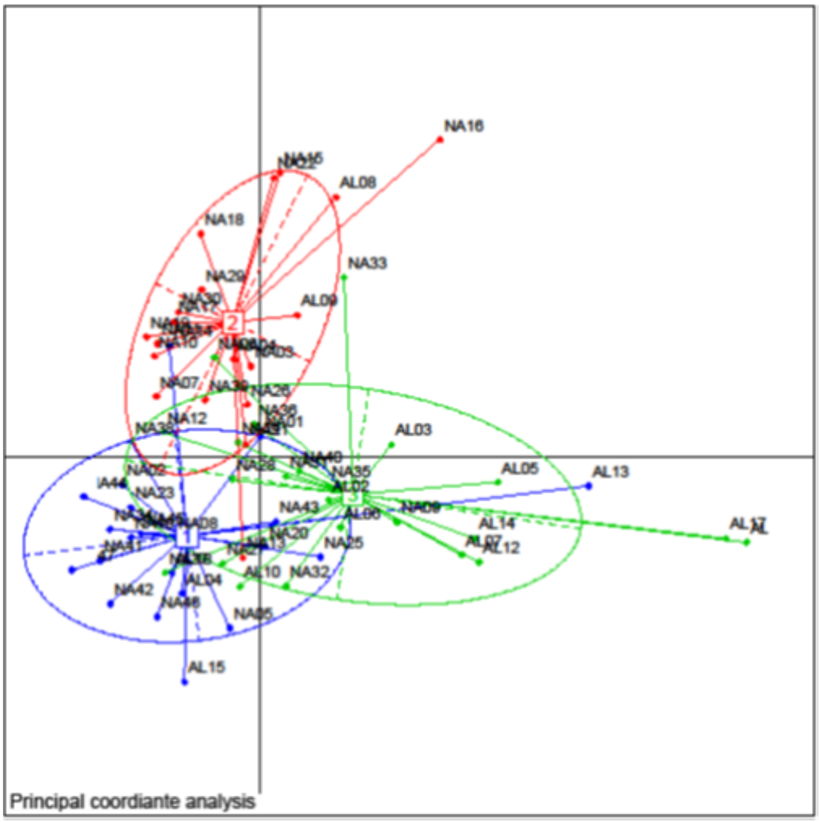

Supplementary Figure S5 (continued on the following page)

(B)

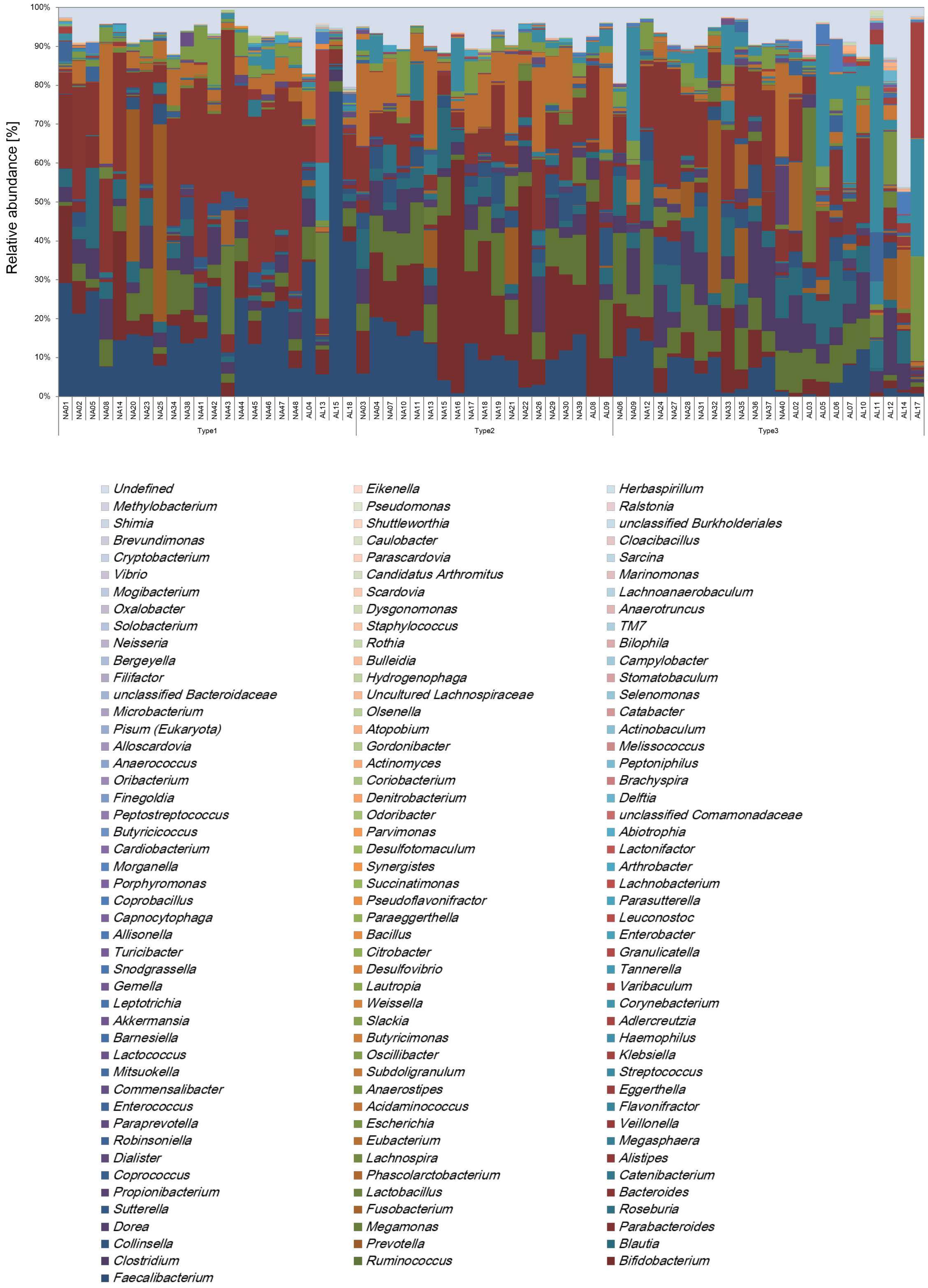

Supplementary Figure S5 (continued on the following page)

（Continued from the previous page）

Supplementary Figure S5 | Enterotyping of GM structures. (A) Identification of three possible enterotypes (termed types 1, 2, and 3) as analyzed by using R. (B) Clustering of GM according to enterotypes. Enterotyping of 64 GM datasets obtained in this study were performed on the basis of relative genus abundance. The analysis allowed us to identify three possible enterotypes [type 1 (*blue*, 22 subjects), type 2 (*red*, 19 subjects), and type 3 (*green*, 23 subjects); see (A)], wherein alcoholic GM structures were distributed unevenly (type 1, 4 subjects; type 2, 2 subjects; and type 3, 10 subjects; see (B)). For comparison, the numbers of non-alcoholic GM structures in types 1, 2, and 3 were almost comparable with each other (18, 17, and 13 subjects, respectively) (B). These results suggest that these three enterotypes could be, primarily, equally identified among non-alcoholics, and habitual drinking (or combined habitual drinking and smoking) might result in enrichment of type-3 GM structure in alcoholics. Consistently, distributions of the subjects with drinking habits (i.e., Group 3) or those with drinking and smoking habits (i.e., Group 4) were also uneven among these enterotypes (see Fig. 3).

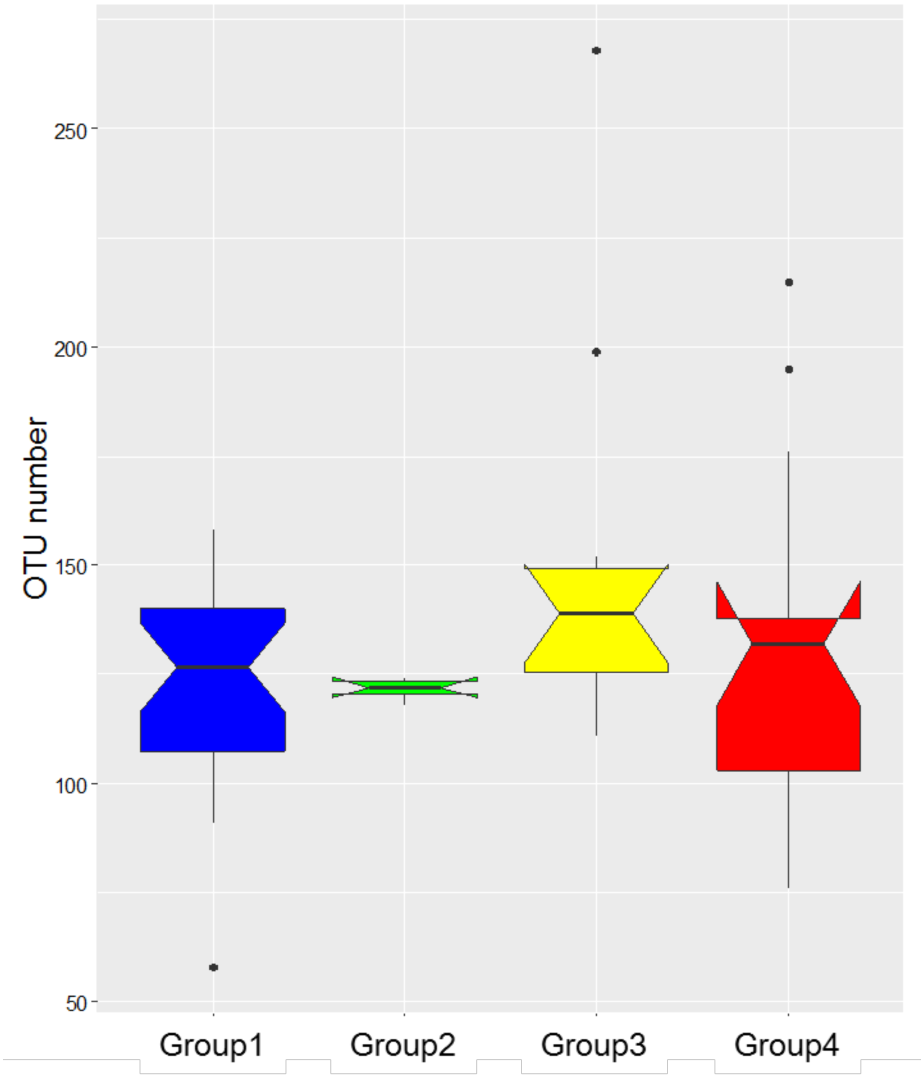

Supplementary Figure S6 | Notched box plots of fecal OTU numbers (α-diversity) of fecal bacterial communities of Groups 1 through 4 classified according to drinking and smoking habits.

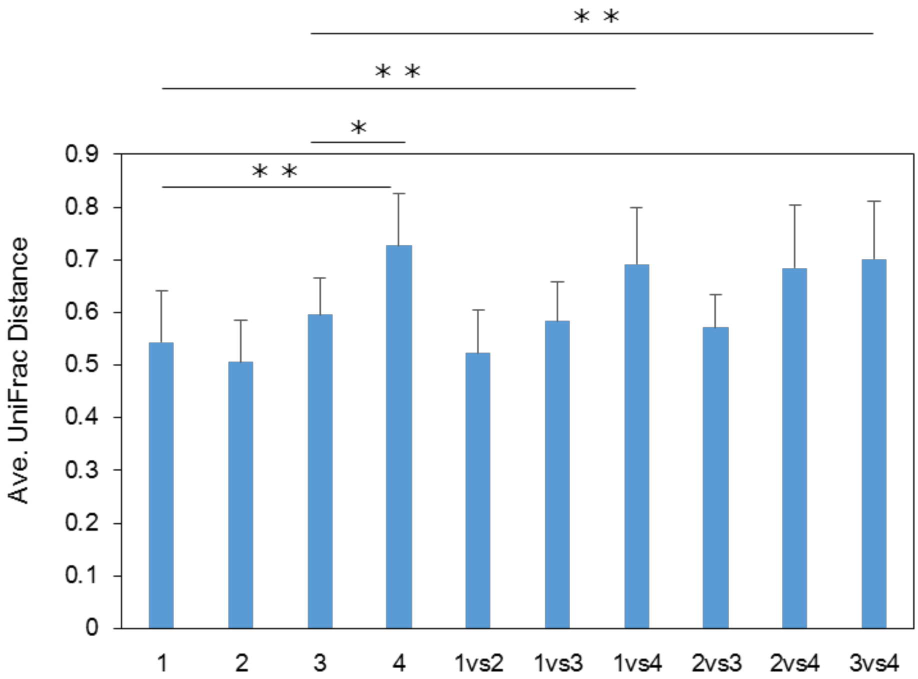

Supplementary Figure S7 | Weighted average UniFrac distances of GM structures determined within a Group and between Groups. Groups 1 through 4 were defined as described in the **Methods** section. *P*-values (by the Welch's *t*-test with 10,000 Monte Carlo simulations) adjusted based on the Bonferroni procedure were $^*P < 0.05$ and $^{**}P < 0.01$.

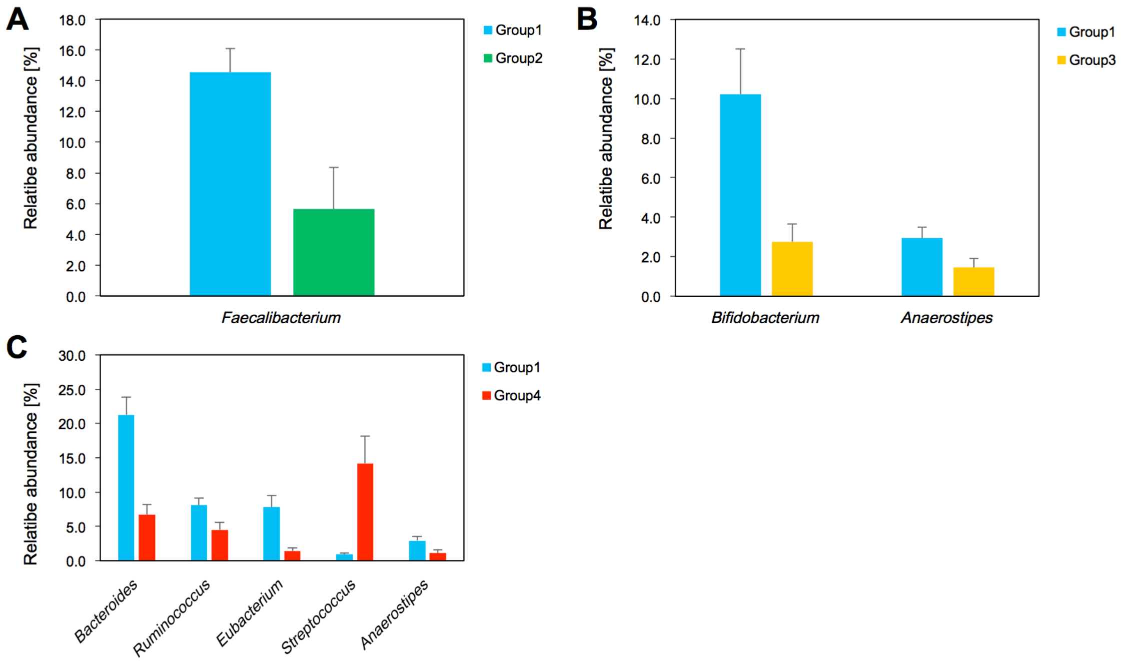

Supplementary Figure S8 | Relative abundances of bacterial groups showing significant differences (*P* < 0.05 according to Welch-test) as compared between fecal bacterial communities of (A) Groups 1 (*blue bars*) and 2 (*green bars*), (B) Groups 1 (*blue bars*) and 3 (*yellow bars*), and (C) Groups 1 (*blue bars*) and 4 (*red bars*).

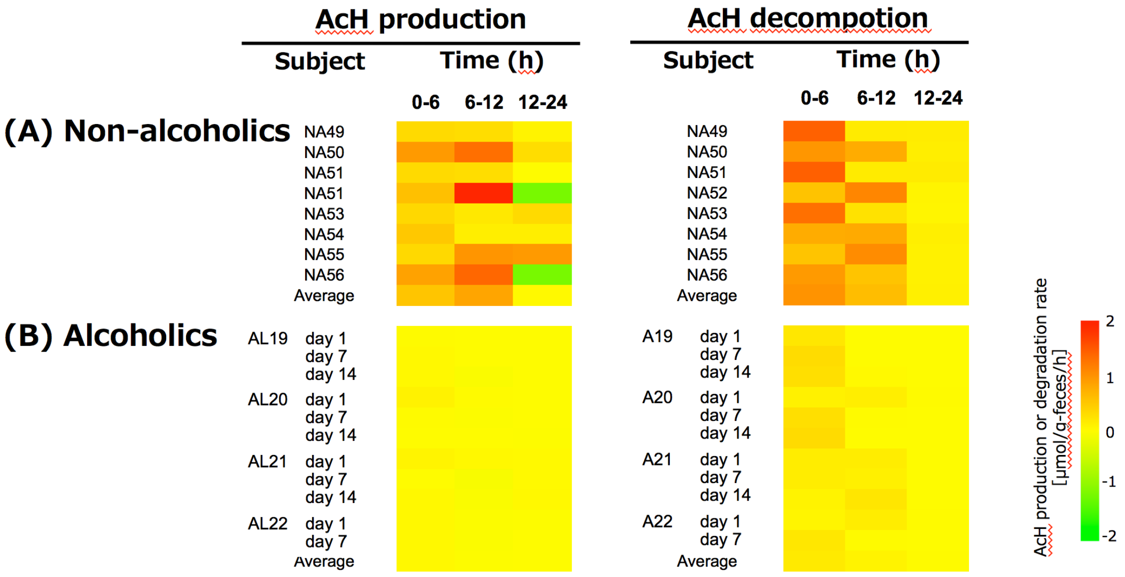

Supplementary Figure S9 | Heat-map representation of the rates of fecal AcH production and decomposition as compared between the alcoholic and non-alcoholic subjects. Rates of AcH production from 22 mM ethanol (*left panels)* and decomposition of AcH (initial concentration, 170 ± 35 µM; *right panels*) during 0–6 h, 6–12 h, and 12–24 h of the reaction by fecal samples of eight non-alcoholic subjects (A) and four alcoholic patients (B) are shown. For experimental details, see the **Methods** section.
